# The Interplay between Wnt Mediated Expansion and Negative Regulation of Growth Promotes Robust Intestinal Crypt Structure and Homeostasis

**DOI:** 10.1371/journal.pcbi.1004285

**Published:** 2015-08-19

**Authors:** Huijing Du, Qing Nie, William R. Holmes

**Affiliations:** 1 Center for Complex Biological Systems and Department of Mathematics, University of California Irvine, Irvine, California, United States of America; 2 Department of Physics and Astronomy, Vanderbilt University, Nashville, Tennessee, United States of America; University of Surrey, UNITED KINGDOM

## Abstract

The epithelium of the small intestinal crypt, which has a vital role in protecting the underlying tissue from the harsh intestinal environment, is completely renewed every 4–5 days by a small pool of stem cells at the base of each crypt. How is this renewal controlled and homeostasis maintained, particularly given the rapid nature of this process? Here, based on the recent observations from *in vitro* “mini gut” studies, we use a hybrid stochastic model of the crypt to investigate how exogenous niche signaling (from Wnt and BMP) combines with auto-regulation to promote homeostasis. This model builds on the sub-cellular element method to account for the three-dimensional structure of the crypt, external regulation by Wnt and BMP, internal regulation by Notch signaling, as well as regulation by internally generated diffusible signals. Results show that Paneth cell derived Wnt signals, which have been observed experimentally to sustain crypts in cultured organs, have a dramatically different influence on niche dynamics than does mesenchyme derived Wnt. While this signaling can indeed act as a redundant backup to the exogenous gradient, it introduces a positive feedback that destabilizes the niche and causes its uncontrolled expansion. We find that in this setting, BMP has a critical role in constraining this expansion, consistent with observations that its removal leads to crypt fission. Further results also point to a new hypothesis for the role of Ephrin mediated motility of Paneth cells, specifically that it is required to constrain niche expansion and maintain the crypt’s spatial structure. Combined, these provide an alternative view of crypt homeostasis where the niche is in a constant state of expansion and the spatial structure of the crypt arises as a balance between this expansion and the action of various sources of negative regulation that hold it in check.

## Author Summary

The small intestinal epithelium, like our skin, is constantly being renewed. In the intestine however, this epithelium is exposed to the harsh digestive environment, necessitating much more rapid renewal. Remarkably, the entire epithelium is renewed every 4–5 days. This raises the question, how can the size and structure of this tissue be maintained given this pace. Motivated by recent experimental observations, we construct a three-dimensional, hybrid stochastic model to investigate the mechanisms responsible for homeostasis of this tissue. We find that there are redundant signals created by both the epithelium itself and surrounding tissues that act in parallel to maintain epithelial structure. This redundancy comes at a price however: it introduces the possibility of explosive stem cell population growth. Additional results suggest that other signals along with choreographed motion of cells are responsible for repressing this expansion. Taken together, our results provide a novel hypothesis for how robust but fast renewal of the crypt is achieved: as a balance between expansion, which drives fast renewal and repression, which holds that expansion in check to maintain the crypt’s structure.

## Introduction

Stem cells have critical physiological roles in both the renewal of healthy tissues and the repair of damage. Intriguingly, while these cells perform the same basic processes as other cells, e.g. growth and division, they are typically associated with a special environment, a “niche”. A common hypothesis for the functional role of such an environment is the regulation of homeostasis [1]. One generic model of homeostatic regulation is the so-called “hand of God” model where external signals regulate stem cell activity. In the intestinal crypt for example, external Wnt signals provided by surrounding tissue have been shown to regulate differentiation [2,3]. An alternative (but not exclusive) possibility is that stem cells build a niche where internal feedbacks as well as feedbacks between the niche and its environment regulate homeostasis. Stem cells in the olfactory epithelium for example have been shown to interact with their progeny and environment through a complex set of diffusible signals to regulate their own population [4]. Similarly, interactions between stem cells of the hair follicle and their progeny are responsible for the predictable timing of cyclic hair growth [5]. Here we investigate how highly local (e.g. at the length scale of a single cell) niche signaling influences the spatial structure of the intestinal crypt and the homeostatic balance between expansion and repression of stem cell populations.

The epithelium of the intestinal crypt is an incredibly dynamic tissue, constantly replenishing itself every 4–5 days. This test tube shaped invagination of the intestine is spatially configured with a proliferative compartment at its base with a compartment of differentiated cells above it that perform various physiological functions critical to digestion. The source of this constant replenishment, like with other organs and tissues, is a small pool of cycling intestinal stem cells (ISCs). Early investigations implicated so called “+4” cells (so named for their position 4 cells up from the base) as the ISCs [6]. Alternatively, it was suggested that crypt base columnar cells (CBCs) interleaved with Paneth cells at the crypt base were the true ISCs [7,8]. These investigations however relied on the Lgr5 marker to indicate stem-ness and a functional approach has suggested that only a subset of these Lgr5 cells are actively participating in crypt maintenance at any given time [9]. A more recent theory has suggested there are in fact two populations of ISCs, active CBCs that steadily renew the crypt and quiescent +4 cells that activate and regenerate it after injury [10,11]. While the debate about the true identity of ISCs remains, it is clear that the CBCs (or some subset of them) at the base of the crypt are responsible for the continual renewal of the crypt epithelium.

This constant replenishment is fueled by approximately 15 CBCs [12]. In contrast to canonical renewal processes however, these CBC stem cells divide exclusively symmetrically [13,14] and that differentiation is decoupled from division [15]. Furthermore, they do so considerably more quickly than in other tissues, dividing roughly once per day even in healthy tissue [16]. How then is homeostasis of such a dynamic tissue maintained? Numerous investigations have shown the canonical Wnt / β-catenin pathway to be critical in maintaining homeostasis [2,3]. This pathway, which regulates gene transcription and cell fate specification, is required to prevent differentiation of stem cells and maintain the crypt. This is evidenced by the complete depletion of stem cells upon disruption of this pathway [17,18].

Interestingly, there are two sources of Wnt signaling in the crypt [2]. The mesenchyme that surrounds it produces graded expression (highest at the base) of a number of Wnts including Wnt2b, Wnt4, and Wnt5a. Additionally, Paneth cells, which are interleaved with the CBCs at the base and commonly referred to as niche cells, also produce Wnt3a. Surprisingly, genetic deletion of this “local”, Paneth cell derived Wnt source does not impair stem cell populations in the *in vivo* crypt [19], suggesting the global Wnt gradient is sufficient for homeostasis. However, i*n vitro* studies of “mini-guts” grown from CBCs have shown that Paneth derived Wnt3a alone is also sufficient to maintain crypt structure in the absence of the other exogenous Wnt sources [20,21].

While Wnt signaling is crucial to crypt homeostasis, there are other important regulatory pathways that are also required. Notch lateral inhibition creates a toggle switch that leads to the salt and pepper organization of stem / Paneth cells in the base of the crypt. This pathway is also responsible for a similar arrangement of secretory (Goblet) and absorptive (enterocyte) lineages further up the crypt walls [22,23]. This salt and pepper arrangement in particular is critical in maintaining the niche structure at the crypt base since contact with a Paneth cell is required to prevent stem cell differentiation [24]. Additionally, Eph / ephrin signaling interactions generate repulsive forces that drive Paneth cells to migrate down the crypt wall while all other cells passively migrate upward from the base, driven by proliferative pressure [25]. Bone morphogenic proteins (BMPs), which form a gradient opposing that of Wnt [26,27], are also known to influence crypt homeostasis by suppressing proliferation of stem cells [28], and their inhibition leads to crypt fission [29].

How do these signaling components contribute to maintaining the spatial structure of the crypt and how do they interact? In addition to experimental interrogation, extensive computational modeling has been employed to address this and related questions. Using optimal control theory, it was shown that a “bang bang” growth process is responsible for crypt formation [30]. Numerous compartment models, which consider the crypt to be spatially well mixed and focus on temporal dynamics, have been used to investigate the processes that promote homeostasis and drive tumorigenesis in crypts [31–34], see [35] for a recent review. Continuum spatial models have similarly been used to investigate the formation and regeneration [26] of crypts as well as mutation acquisition [36] in them.

Each intestinal crypt however contains on the order of tens of stem cells and hundreds of total cells and is thus a highly stochastic entity. Further, the spatial arrangement of stem and Paneth cells at the crypt base has an important role in niche homeostasis. Discrete models accounting for individual cell dynamics and interactions have been developed to account for these features. In [37], it was shown that the geometry of the crypt could affect organ aging and susceptibility to cancer. Wong et al. [38] demonstrated that under certain conditions, Eph / ephrin mediated differential adhesion is required for proper crypt organization. In [39,40], discrete modeling methods were used to show that the basement membrane has a critical role in defining the crypt geometry, which is crucial for proper function. In [41], the geometry was further shown to have a significant impact on the time it takes for neutral drift to drive a crypt to mono-clonality, which has implications to mutation acquisition and fixation. In [42,43], it was shown that Wnt and Notch signaling are critical to organizing crypt architecture and that under the assumption of reversible cell fate specification, this architecture is extremely robust to perturbation. Extensive use of agent based modeling [44] has been used in this domain as well. Bravo et al. [45] constructed a 2D agent-based crypt model that was calibrated to human biopsy data to accurately account for the number of cells of different types as well as the variance of those numbers. They then used this as an *in silico* test platform to determine the efficacy of different cancer therapy protocols. For further review of the extensive discrete crypt modeling literature, see [46].

Most of these investigations have however been directed at understanding the physical structure of the crypt and how it influences function, rather than the role of niche signaling on homeostasis. Those that have incorporated signaling have thus far primarily focused on the influence of external signals, exogenous Wnt signaling in particular [42]. Here, we extend these investigations to investigate the role of local production of Wnt by Paneth cells as well as negative regulation via BMP. Toward this end, we build a comprehensive discrete model accounting for both the physical structure of the crypt and these signaling interactions. This model is then probed to determine the influence of these different signaling components and the implications of their deletion.

A number of 2D [38,47] and 3D [42,48] models have been utilized to investigate various aspects of crypt dynamics in the past. Here, we utilize the relatively new sub-cellular element method (SSEM) [49,50] to treat individual cells as discrete, deformable objects. The SSEM provides a natural framework to describe the mechanical force interactions between cells, which is important in this application. This method has been previously employed to model both multi-cellular systems and single cell dynamics [51]. In the multi-cellular context, it was used to describe the dynamics of epithelial sheets [49], primitive streak formation in the chick embryo [52], the influence of Notch signaling on regulatory networks controlling cell division [53], the dynamics of bacterial swarms [54], and the dynamics of epithelial layer formation [55]. Here we use this framework to construct a model of the 3D, dynamically evolving crypt. Within this model, we account for cell-cell force interactions, Ephrin mediated repulsion of Paneth cells, cell-cell Notch signaling, exogenous Wnt and BMP signaling, as well as local Paneth cell derived Wnt signaling. The latter of which requires a substantial augmentation of the SSEM to account for the presence of diffusible signals.

By interrogating this model computationally, we show that indeed, multiple sources of Wnt signaling can act redundantly to maintain the crypt. A crucial implication of this redundancy however is that the stem cell niche is in a constant state of expansion, which if left unchecked would lead to the niche cannibalizing the entire crypt. Further results however indicate that inhibition of proliferation by BMP can constrain this expansion and promote homeostasis. This view also points to a different interpretation of the function of downward motion of Paneth cells. We find that this motion is not required to maintain the niche as might be expected (given the role of Paneth cells in niche signaling), but rather it is again needed to constrain niche expansion. Taken together, these results suggest that different Wnt sources have significantly different influences on niche homeostasis, and that negative regulation is required to balance the expansive influence of Paneth derived Wnt signaling.

## Results

### Model description

Here we describe a three-dimensional, multi-scale model of the dynamically evolving crypt. This model combines 1) a subcellular element formalism describing physical / mechanical aspects of cellular dynamics, 2) a chemical diffusion-reaction model for endogenously produced signaling, and 3) a stochastic differential equation model of lineage regulation for each individual cell. For further details, see Materials and Methods. For all parameters listed, see Table 1.

**Table 1:** Listing of simulation parameters for this model. In cases where parameters are drawn from literature, references are provided.

Parameters	Values
Cell diameter	10 microns [13,42]
Crypt height	160 microns
Crypt diameter	60 microns
Cell division cycle	24 hours [56]
Element number (*N*)	20
Intra-cellular potential	*μ* = 2.5,*r*_0_ = 1.5, no cut-off distance.
Inter-cellular potential	*ε* = 0.05,*σ* = 4.5, cut-off distance is 10.0.
External force	*ε*_*external*_ = 0.001, cut-off distance is 5.0.
Linear drag in z-axis (*b*_*z*_)	-0.3
Time step (dt)	3.6s for SSEM; 0.0036s for chemical equation
Wnt threshold (*TH*_*Wnt*_)	0.85 [42]
BMP threshold (*TH*_*BMP*_)	0.15 [42]
Notch activation (*NP*)	0.35 for Paneth cell; 1.0 for Goblet cell [42]
Wnt production (*δ*_*c*_)	0.01
Baseline Diffusion (*D*)	10^−7^ *cm* ^2^ / *s* [62]
Decay (*d*)	10^−3^ / *s* [62]

### Cellular dynamics

We utilize an extension of the subcellular element method to describe each discrete cell of the evolving crypt as a deformable object. In this formulation, a cell is described by a collection of *N* sub-cellular elements [49] that interact pairwise according to user defined forces. These forces encode short-range repulsion, which endows each element with a “volume”, and medium-range attraction, which causes all elements to form a coherent cell. In the absence of external forces, energy minimization will cause these cells to round up to a preferred spherical shape and volume. Additionally, we model direct cell-cell interactions by specifying forces between different cells that mimic contact and adhesion. The resulting model forms a large system of differential equations that describe the evolution of all elements (and hence the cells themselves) in time. The flexibility of this method further allows specification of different properties based on a cell’s identity. One such difference we include is the tendency for Paneth cells to move downward toward the base of the crypt, while all other cell types move passively up the crypt wall in response to proliferative pressures. See Materials and Methods for further details.

In addition to cell-cell interactions, forces between each cell and its environment are also prescribed. Each cell is assumed to adhere to a rigid, test tube shaped basement membrane. Additionally, a drag force between cells and the membrane is imposed, mimicking the friction caused by the need to break and re-form bonds as the cells move. When cells reach the upper bound of the crypt domain, they are removed. Similarly, if detachment of a cell from the basement membrane is detected, it is removed.

### Cell types

**Fig 1:**
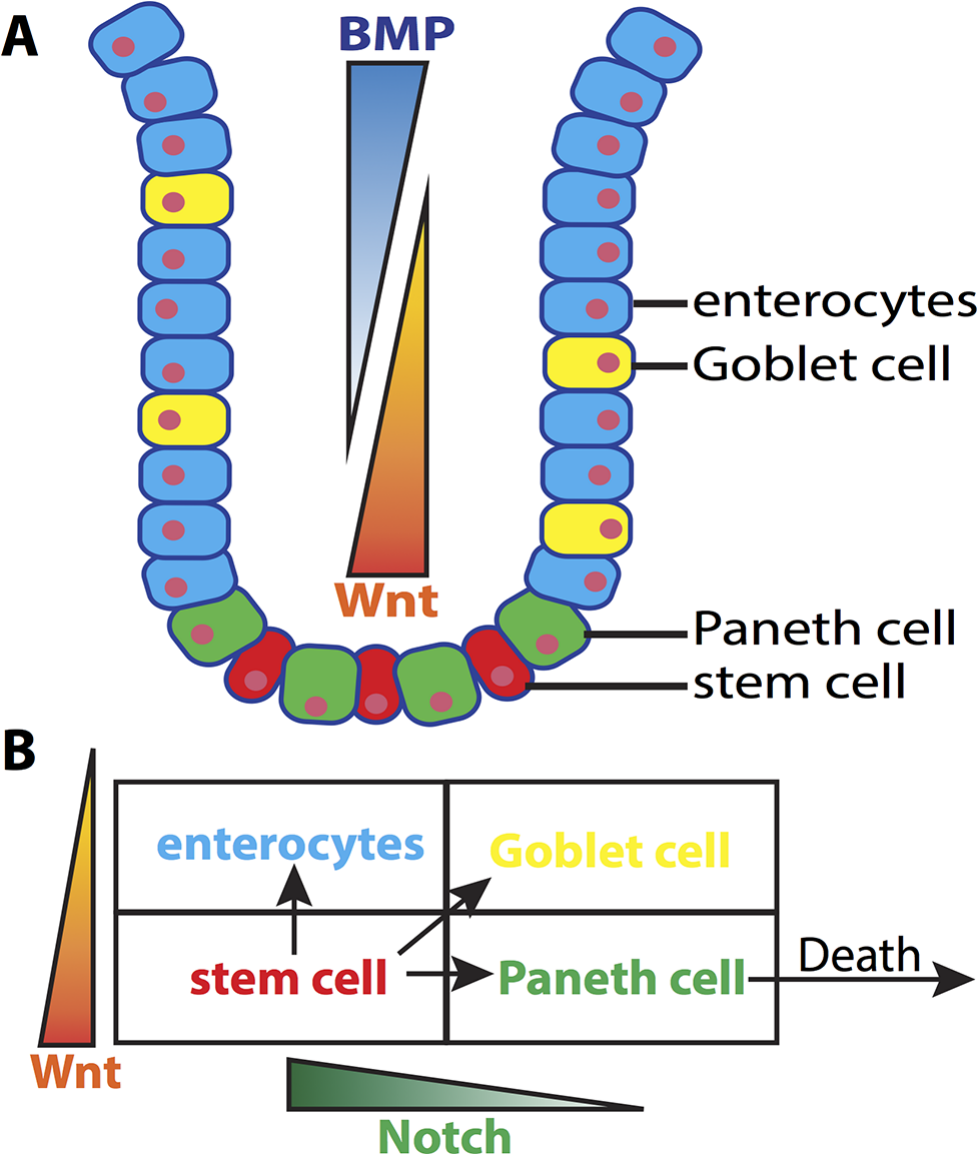
A schematic illustration of the crypt’s structure and cell lineage classification. ***Panel A)*** A cartoon of the intestinal crypt and the relative location of cells of different lineages. ***Panel B)*** A diagram of cell lineages. Stem cells give rise to terminally differentiated enterocytes, Goblet and Paneth cells, depending jointly on Wnt concentration as well as Notch expression of that cell’s neighbors. Only Paneth cells undergo apoptosis, since Goblet cells and enterocytes naturally undergo anoikis upon reaching the top of the crypt.

Four primary cell types are considered: stem, Paneth, enterocyte, and Goblet. The former two are well known to occupy the base of the crypt while the latter two comprise the upper crypt epithelium (Fig 1A). Since we are primarily interested in how signaling in the stem cell niche influences crypt dynamics and stability, we simplify the system by assuming only stem cells are proliferative and do not include the transient period during which enterocytes and Goblet cells further up the crypt divide. Following a previous study [42], we assume Wnt- and Notch-signaling jointly regulate fate specification (Fig 1B). For both pathways, we assume cells above or below the threshold (*TH*_*Notch*_ for Notch and *TH*_*Wnt*_ for Wnt) take different fates. *I*_*Notch*_ denotes the notch activation level of a cell, which is determined by the activation of its neighbors. *I*_*Wnt*_ represents the Wnt level that a cell is exposed to, which is the sum of contributions from the external Wnt gradient and the Wnt produced by nearby Paneth cells. Fig 1B indicates the combination of these signaling levels that determine each cell’s fate. As in [42], we assume that cells can reversibly transition between Goblet and enterocytes fates, depending on Notch levels. Paneth cells are further assumed to terminally differentiate, after which they enter a long-lived quiescent state.

### Wnt signaling

We assume two sources of Wnt influence cell differentiation: a global gradient derived from the surrounding mesenchyme, and an additional contribution being produced by Paneth cells in the niche. The global gradient is assigned to be highest at the crypt bottom and to decrease gradually along the crypt axis. Given the lack of *in vivo* concentration information and the fact that the relative levels of a morphogen determine the spatial cues, this quantity is non-dimensionalized in the range [0,1]. Initially, we will assume this gradient is deterministic but later will consider the influence of noise superimposed on that gradient, which we assume to be uncorrelated (in space and time) multiplicative noise. Each Paneth cell is further assumed to secrete Wnt at a constant rate. This signal field is modeled using the chemical reaction-diffusion equations:

$$\frac{\partial c}{\partial t}=D_{c}\Delta c+\delta_{c}n_{Paneth}-d_{c}c,$$ (1)

where *c* denotes Wnt concentration at a given location in space and time. The second term on the right hand side *δ*_*c*_ *n*_*Paneth*_ represents the total secretion rate, *δ*_*c*_ is the secretion rate of an individual Paneth cell, and *n*_*Paneth*_ is a measure of the local density of Paneth cells at each grid location. This mapping is used to spread the production of each Paneth cell over the grid nodes that each Paneth cell occupies. *D*_*c*_ is the diffusion coefficient, and *d*_*c*_ is the decay rate. For simplicity, we assume that the signal cannot diffuse across the basement membrane or into the crypt lumen. Rather than define no flux boundary conditions on complex surfaces, we instead extend the computational domain beyond the domain containing the cells and assign the chemical diffusion coefficient to be *D*_*c*_ = 0 on the extended domain. In addition to simplifying boundary conditions, this also allows the use of a box shaped domain, which is simpler computationally. This chemical field is simulated on the regular grid, and a reverse mapping is used to determine the value of this Wnt field that each cell is exposed to. The contribution of the global Wnt gradient is then added to this local value to generate the total Wnt field.

### Notch signaling

The Notch activity is calculated via direct cell-cell contact analysis. A cell is Notch-activated by direct neighboring cells expressing Notch-ligands according to

$$I_{Notch}=\sum_{cell}\delta (i)NP.$$

Here, the sum runs over all neighboring cells of the target cell. *δ*(*i*) is equal to one if cell *i* is in contact with the target cell (which is determined by proximity), otherwise it is zero. The degree of activation by a single cell (*NP*) depends on the cell type. *NP* is assumed to be larger than zero for Paneth and Goblet cells and zero for all other cells. A cell changes its fate if its Notch-activity crosses the threshold *TH*_*Notch*_.

### BMP signaling

We include the influence of BMP signaling on cell proliferation. Similar to Wnt gradient, a global BMP gradient is applied along crypt axis. This gradient opposes the Wnt gradient however, with low levels at the base that increase as you move up the crypt (again in the range of [0,1]). Multiplicative noise is again applied to mimic the stochasticity of this gradient. Threshold regulation is similarly assumed, so that if a cell is exposed to BMP levels above *TH*_*BMP*_, proliferation is inhibited.

### Growth and division

Cells grow at a constant rate; an element is added to each cell at regularly scheduled intervals, resulting in a volume increase. Only stem cells are capable of proliferation, with a cell cycle of approximately 24 hours [56]. This is taken as the mean value of a normal distribution (with standard deviation of 4 hours), truncated to the interval 20–28 to ensure reasonable values. Each cell is assigned a division cycle length and an internal timer. At beginning, the internal timer is set to zero, and increments every time step. If the inner time counter exceeds the assigned division cycle length, that cell undergoes division. When division occurs, a plane perpendicular to the crypt wall with a randomly chosen angle is assigned as the division plane to split the cell. This is done to ensure both daughter cells maintain contact with the wall after division. The elements of the cell are then divided so that the daughter cells have an equal number of elements (plus or minus one). For each daughter cell, the internal clock counter is set to zero. Paneth cells are assumed to enter a quiescent phase after differentiation. We thus assume that after differentiating, each Paneth cell undergoes apoptosis. The lifetime of Paneth cell is normally distributed with mean 8 weeks [21] and standard deviation 2 weeks (this distribution is truncated to the 6 to 10 week range to ensure reasonable values are chosen).

### Simulation results

#### Paneth derived Wnt promotes uncontrolled expansion of the stem cell niche

It is well established that crypt homeostasis is at least partly regulated by a gradient of mesenchyme derived Wnt, which via the canonical pathway prevents stem cell differentiation. While it is known that this external or “global” Wnt signal is involved in homeostasis, recent evidence suggests that Paneth [2,20] cell derived Wnt signaling may have an important role as well. *In vitro* cultures, where morphologically correct, functional “mini guts” (e.g. crypt / villus structures) are grown from a single stem cell, have demonstrated that stem cells co-cultured with Paneth cells are much more likely to form organoids than those cultured alone [20,21]. Further, the stem cells in the resulting crypts are maintained in the complete absence of Wnt derived from surrounding tissues. Accordingly, we first ask how Paneth derived Wnt signaling influences stem cell niche structure and stability.

Based on a previous modeling work [42], we construct a 3D model of the crypt (see Materials and Methods for details) accounting for growth and division of cells, cell–cell interactions, Notch lateral inhibition, and the presence of an external graded Wnt signal that, when above a threshold, prevents differentiation of stem cells. This core model (with parameterization discussed in Materials and Methods), leads to the development of a stable crypt with a stem and Paneth zone roughly four cells in height occupying the base, consistent with observations. To investigate the influence of additional Wnt signaling, each Paneth cell is further considered to be the source of a second, “local” and diffusible Wnt source with rates of diffusion taken from [26]. Since Wnt concentration, rate of production, and degradation rate are not available, we chose a base set of these remaining parameters so that the Wnt concentration one cell diameter from the production source equals the critical Wnt threshold for differentiation.

**Fig 2:**
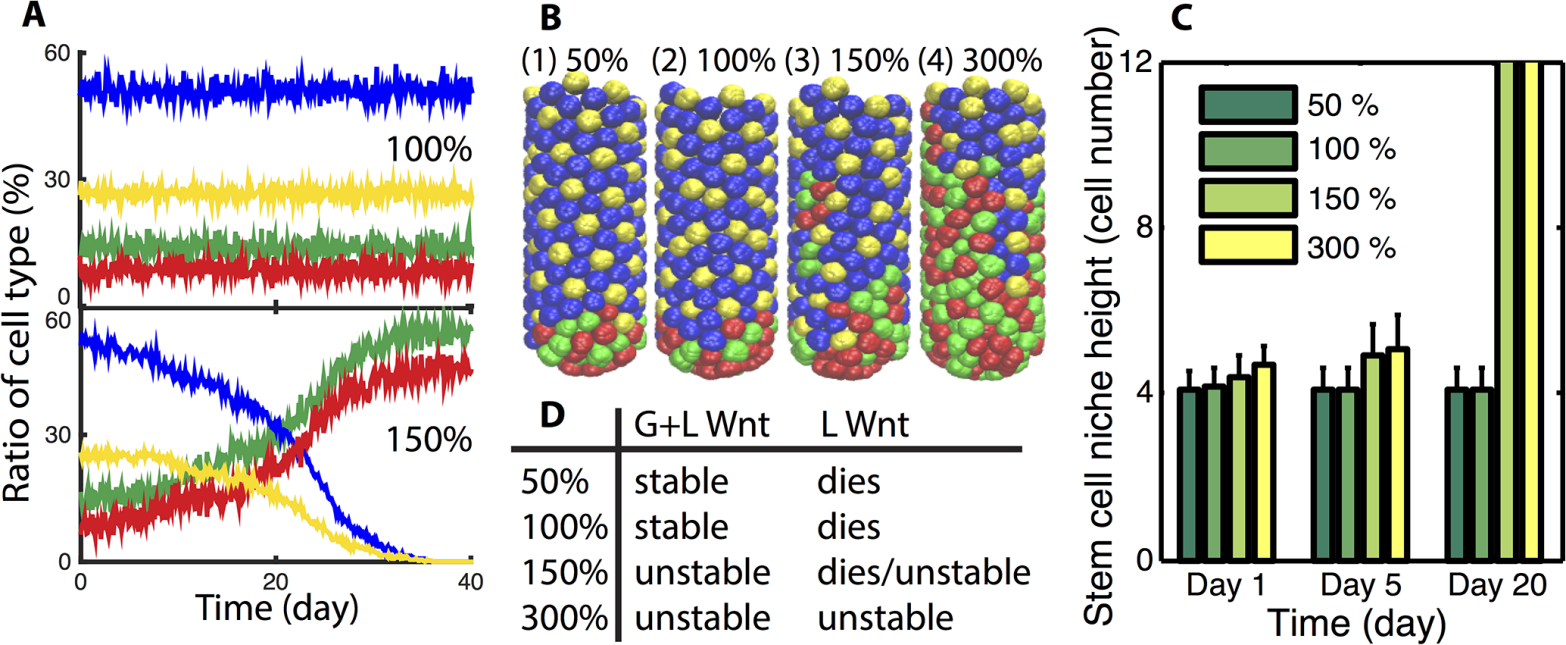
Additional local Wnt production by Paneth cells leads to stem cell niche expansion. ***Panel A)*** Ratio of each cell type of a typical crypt when local Wnt production capability is 100% and 150% for each Paneth cell, respectively. Color code for Panel A and Panel B: stem cell (red), Paneth cell (green), enterocytes (blue) and Goblet cell (yellow). ***Panel B)*** Snapshots of crypts at day 10 for different local Wnt production levels. ***Panel C)*** Plot of niche height as a function of different Wnt production rates at multiple times. Mean and standard deviation of an ensemble of 10 simulations is reported. Cases where the bar extends to the top indicate the niche is unstable and expands to occupy the entire crypt. ***Panel D)*** Indication of how the global and local Wnt influence the broad dynamics of the niche. Left column indicates the local Wnt production rates considered. G+L indicates that both global and local, Paneth cell derived Wnt are included while the right (L) column considers the setting where the global Wnt gradient is removed. “Stable” indicates a properly structured, steady state crypt results, “dies” indicates the niche (stem and Paneth cells) is completely lost, while “unstable” indicates the niche undergoes uncontrolled expansion. In no scenario is the niche stable both before and after the removal of the Wnt gradient, indicating in this setting Paneth cell derived Wnt cannot act redundantly.

Simulations are conducted to determine the influence of this added Wnt signaling. Simulations at four different levels of Paneth derived Wnt production are then conducted (Fig 2). All simulation specifics (e.g. initial conditions) are discussed in Materials and Methods. Results indicate that when the local production rate of Wnt is at or below this base rate (100% level), the niche remains stable. However, when that production rate is raised to a level sufficient to sustain stem-ness of neighboring cells, the niche expands and takes over the entire crypt. Time course data and snapshots of the crypt state at a fixed time (Fig 2A and 2B) clearly show the fraction of stem and Paneth cells increase in time at the expense of the fraction of other differentiated cell types. Additionally, an ensemble of simulations (which averages over the inherent stochasticity of these simulations) in Fig 2C indicate that the height of the niche remains stable for low production rates but grows in time for higher production rates.

The essential source of this expansion is a positive feedback between stem and Paneth cells. When the Wnt production rate is sufficiently high, locally produced Wnt alone is sufficient to sustain neighboring stem cells. Those stem cells however produce yet more Paneth cells, creating a mutually sustaining feedback that drives expansion of both populations. Thus if Paneth cell derived Wnt signaling is sufficiently strong to prevent differentiation of neighboring cells on its own, the niche will undergo uncontrolled expansion and homeostasis will not be maintained. If on the other hand, production rates are below that critical threshold (the base 100% level in Fig 2), homeostasis is maintained in normal tissue, but this source is no longer redundant. In this case, if the global Wnt gradient is interrupted, all cells differentiate and the niche is lost (Fig 2D). Thus, Paneth cell derived Wnt signaling alone cannot both fully sustain the niche and promote homeostasis at the same time.

#### Regulation of proliferation by BMP constrains niche expansion

The above demonstrates that levels of Paneth derived Wnt production sufficient to prevent differentiation lead to uncontrolled expansion of the niche. Thus while this redundant Wnt source can help maintain the niche, some other factors are needed to constrain its expansion. BMP, which has an opposing gradient to that of Wnt, is known to suppress proliferation and is thus a potential candidate. To investigate its role in niche stability and homeostasis, we build on the previous model (including the presence of Paneth cell derived Wnt) to include the global BMP gradient and assume that above a threshold, BMP prevents proliferation of stem cells.

**Fig 3:**
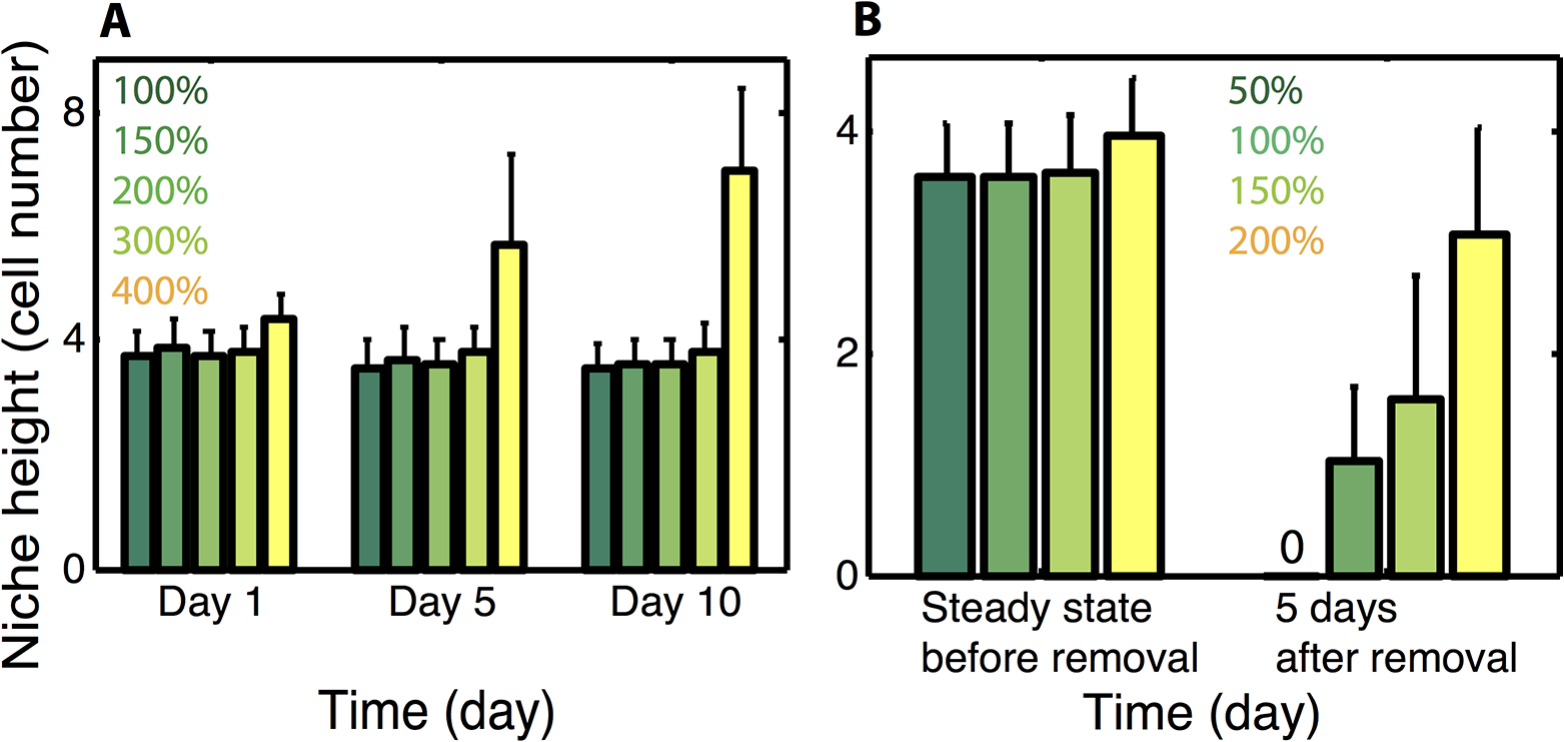
Role of BMP on niche homeostasis. ***Panel A)*** Niche height as a function of different local Wnt production rates with BMP inhibition of proliferation considered. Mean and standard deviation of an ensemble of 10 simulations is presented. For the 400% production level, expansion continues in time until the niche completely overtakes the crypt. For the 100–300% cases, stability of crypt height has been verified with extended simulations. ***Panel B)*** Same as in (A) but with the global Wnt gradient removed. For low production rates, the niche is either completely lost or substantially smaller, but at higher rates (e.g. 200%), the niche height is only slightly impaired, indicating that at these levels the two Wnt sources can function redundantly. In all cases (in B), the niche heights at 5 days post removal represent steady state results.

The crypt is again initialized in a canonical configuration and allowed to evolve under the influence of the combination of the global Wnt gradient, the BMP gradient, and Paneth cell derived Wnt. Given the observed influence of local Wnt production rates, different rates are again considered to determine the efficacy of BMP at constraining growth under different niche expansion conditions. Results (Fig 3A) indicate that for moderate levels of Wnt production, BMP is effective at constraining niche expansion and maintaining homeostasis. Interestingly, with this form of repression, the crypt structure is highly robust and the niche size is insensitive to Wnt production rates. That is, raising the production rate from 100% to 300% of the critical base level has no significant influence on niche height. The exception to this is that at very high levels of Wnt production (400% in Fig 3A), the niche becomes substantially enlarged. These results suggest that BMP inhibition of proliferation is an effective mechanism for repressing crypt expansion and robustly determining the niche size, provided Wnt production rates are not excessively large.

We next return to the question of redundancy and ask how removal of the global Wnt gradient changes crypt structure (Fig 3B). Crypts with a canonical initial configuration are considered with different local Wnt production rates (50~200%). After they reach the steady state, defined by a stable niche height and ratio between different cell types, the global Wnt gradient is removed from the system, and the crypts are analyzed 5 days after signal removal. Results show that when production rates are low (50% level in Fig 3B), all stem cells differentiate and the niche is lost. For rates slightly above that critical rate (150%), stem cells remain but the niche shrinks substantially. At higher levels however (200%), removal of the global Wnt signal has little influence on the niche, which is maintained at nearly the same size as before Wnt deletion.

Combined, these results suggest there is a balance between expansion and repression that is required to maintain homeostasis. Wnt, which influences differentiation, promotes niche expansion while BMP, which influences proliferation, constrains that expansion. This view is consistent with previous observations that removal of BMP signaling leads to crypt fission, which would be one potential outcome of aberrant niche expansion [29]. This separation of function has the added benefit of making the niche highly robust, as the two Wnt sources are completely redundant. The niche and the broader crypt can cope with complete deletion of mesenchyme derived Wnt (Fig 3), with only a marginal influence on the niche size.

#### Eph/Ephrin mediated Paneth cell motion is required to constrain niche expansion

It is well established that Paneth cells migrate toward the crypt base, fighting against the passive flow of the epithelium up the walls [25]. While it is well established that Eph/Ephrin signaling is responsible for this migration [57], it remains unclear what the purpose of this migration is. One common explanation is that this motion is required to maintain the niche, since contact with a Paneth cell is required to prevent stem cell differentiation at the crypt base [24]. Alternatively, it has been suggested [38] this signaling is critical to maintain a distinct border between different zones of the crypt.

**Fig 4:**
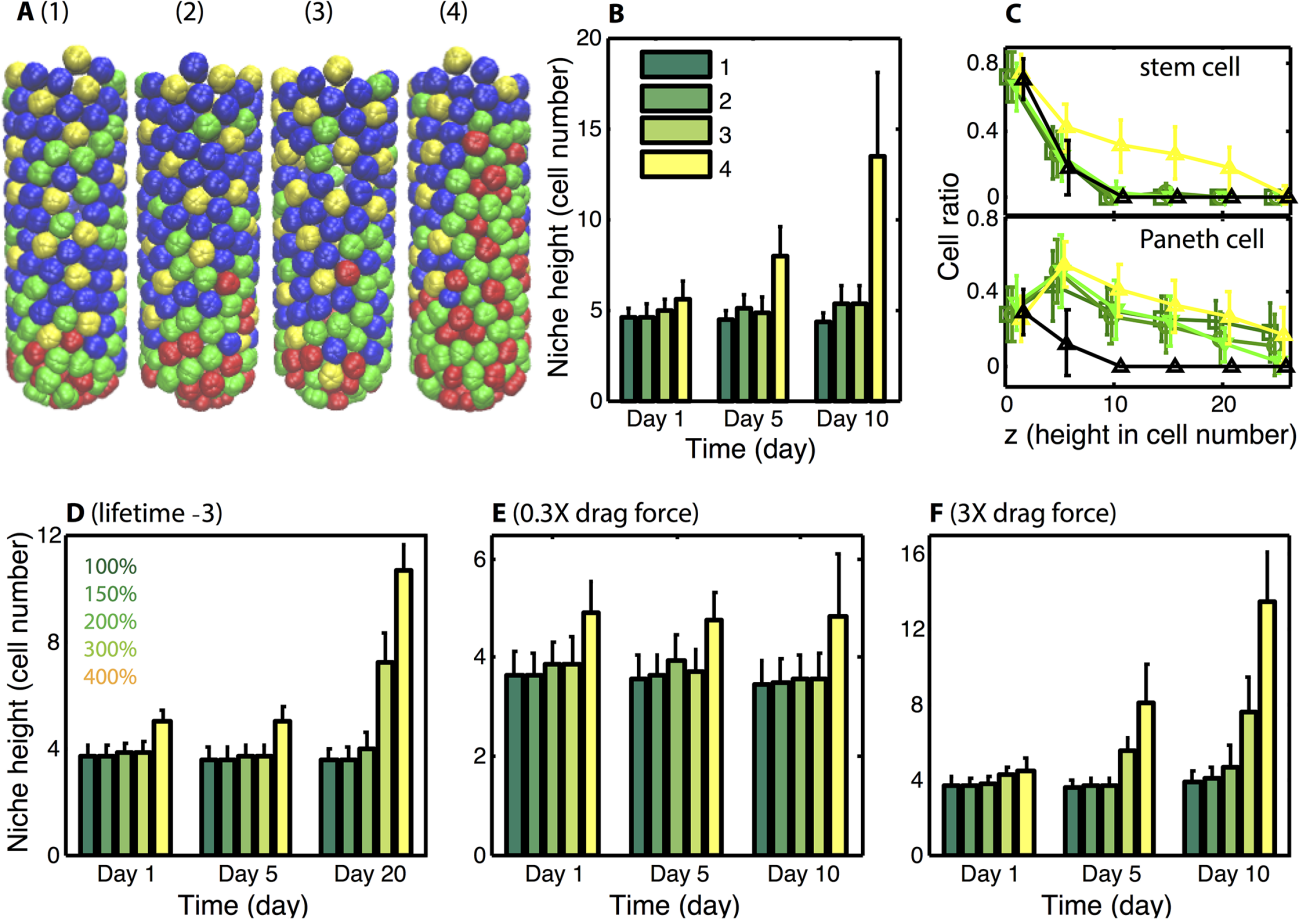
Downward Paneth cell migration is critical for the stability of stem crypt. ***Panel A)*** Snapshots of typical crypts at Day 10 for four models. Color code for Panel A: stem cell (red), Paneth cell (green), enterocytes (blue) and Goblet cell (yellow). In all cases, Paneth cell migration is deleted so that they are subject only to the natural proliferative pressures. Model 1) Only the global Wnt gradient is present. Model 2) In addition to the global Wnt gradient, local Wnt production is included at the 100% level. Model 3) BMP inhibition of proliferation is added to Model 2. Model 4) Global Wnt and BMP gradients along with local Wnt production at the 200% level are included. ***Panel B)*** Results of an ensemble of 10 simulations for each model, niche height is reported at different times. Note that for models 1–3, stem cells are confined to the crypt base. In model 4 however, the stem cell population expands to reach the top of the crypt. In all cases, the Paneth cell population expands to the top of the crypt due to the lack of active migration. The provided color code indicates the model considered. ***Panel C)*** Spatial density of stem and Paneth cell along the z-axis at day 10. Black lines represent the populations for a control model with Paneth migration included (with 100% Wnt production and the BMP inhibition included) and the remaining curves are color coded as in (B). ***Panel D)*** Niche height as a function of different local Wnt production rates (100–400%) with reduced stem cell lifetime considered. Quasi steady state is reached for low local Wnt production rate (100–200%) while unconstrained expansion is observed for 300–400%. ***Panel E-F)*** Niche height as a function of different local Wnt production rates with reduced (E) and strengthened (F) drag force considered. The niche is stable for all cases with reduced (0.3X) drag. For enlarged (3X) drag, crypts are stable only for small local Wnt production rate (100–200%).

To determine the role of this migration in crypt homeostasis, we consider four separate models (Fig 4A–4C). In each case, the base model with Paneth migration leads to stable niche formation, and we consider here how deletion of that migration influences homeostasis. In the first, Paneth derived Wnt and BMP signaling are not considered, so that the external Wnt gradient is solely responsible for homeostasis. Under this circumstance, abrogating this downward motion leads to Paneth cells interspersed along the crypt walls, but leaves the stem cell niche unchanged. When Paneth cell derived Wnt is included at low levels (model 2), removal of directed Paneth motion has the same effect. Inclusion of BMP signaling (model 3) again leads to the same results. In the final model (model 4), both BMP signaling and Paneth derived Wnt at levels that are sufficient for redundancy (200%) are included. In this case we see that loss of downward migration leads to expansion of both stem and progenitor cell populations and the niche takes over the crypt.

In all cases, stem and Paneth cells remain at the crypt base after deletion of migration. Thus, this migration is not required for niche maintenance. It is however required to maintain the remainder of the crypt, the villus in particular. In all cases, this deletion leads to an abnormal upper crypt, with Paneth cells interspersed with Goblet and enterocytes. In the redundant model however, this deletion is catastrophic, leading to complete expansion of the niche. These results suggests that rather than being required to maintain the niche, Paneth cell migration is instead required to maintain proper structure in the upper walls of the crypt, and in particular to constrain niche expansion.

Given the importance of Paneth migration on dynamics, we further investigated the role of cell motions. Two general factors will influence the migration dynamics of cells in this system: 1) the rate of cellular proliferation and 2) the drag between cells and the crypt wall induced by adhesion. We vary both of these properties and determine how they influence dynamics by comparing crypt stability to results in Fig 3A (which we consider as a base case).

First, we decrease the cell cycle length of stem cells by three hours (Fig 4D), thus increasing the proliferation rate. In the base model (Fig 3A), only the 400% Wnt production level destabilized the crypt. When the proliferation rate is increased (Fig 4D), the crypt becomes destabilized at lower Wnt production rates (300%). We additionally varied the drag strength between cells and the crypt wall (Fig 4E and 4F) and again compare results to Fig 3A. When the drag force is decreased (Fig 4E), the crypt is stable at all Wnt production rates considered. When it is strengthened (Fig 4F) however, the crypt is again destabilized at lower Wnt production levels (300%).

These results can be traced back to a balance between the conveyer belt like migration of cells flowing up the crypt wall and active Paneth cell migration toward the crypt base. Consider the analogy of a person walking in opposite direction on a moving conveyer belt. If that person walks a little bit faster than the belt, they will progress toward one end (e.g. the crypt base). If they are a little slower than the belt, they will get pushed in the other direction (e.g. the top of the crypt). We thus expect a bifurcation to occur where sufficiently strong motion of Paneth cells (relative to the proliferative pressure) yields stability while weaker motion leads to instability. Increased proliferation rate (Fig 4A) would in a sense speed the conveyer belt, having a destabilizing effect if Paneth properties were unchanged.

In the absence of contact dependent inhibition of proliferation (which we do not consider), the rate of passive cell migration up the crypt wall will not depend on the drag strength since the rate of cell production at the base must equal the rate of cell removal at the top. It will however have an influence on the active motion of Paneth cells. That is, a stronger drag or adhesion would tend impede active Paneth migration, which would be akin to putting glue on the person’s shoes in the analogy. This explains why larger drag yields crypt expansion while smaller drag leads to crypt stability. We do however note that contact dependent inhibition of proliferation could have an influence on these results since it would place a maximum on the proliferative pressure at the base of the crypt. Jointly, these results do however suggest that the dynamics of Paneth migration relative to the passive motions of other cell types have a strong influence on homeostasis, and that rather than being necessary for niche maintenance, Paneth migration is instead required for niche stability.

#### The length scale of Wnt diffusion has a critical effect on crypt homeostasis

It has known that in the crypt, Wnt signaling above a threshold concentration suppresses stem cell differentiation. Thus it is the concentration field generated by each Paneth cell that is critical in determining its ability to suppress differentiation and contribute to crypt dynamics. While geometry and the cellular neighbor arrangement (i.e. how many Paneth cells surround a stem cell) will influence the concentration field produced by all Paneth cells, we can elucidate the influence of Wnt properties on homeostasis by considering the field generated by a single cell. Consider the simplified setting of a single Wnt generating Paneth cell placed at *x* = 0 in the one-dimensional domain [0,∞]. Suppose further that the Wnt production rate of such a cell is *P* and that the diffusion field generated by this source evolves according to only diffusion and decay

$$W_{t}=DW_{xx}-\delta W,\;-DW_{x}(0)=P,$$

where *D* is the rate of diffusion, *δ* is the rate of degradation, and the latter of these specifies the flux, or rate of Wnt production at the cells location. The resulting steady state concentration field generated by this Paneth cell is then

$$W_{ss}(x)=\frac{P}{\sqrt{\delta D}}\exp\left(-\sqrt{\frac{\delta }{D}x}\right).$$

**Fig 5:**
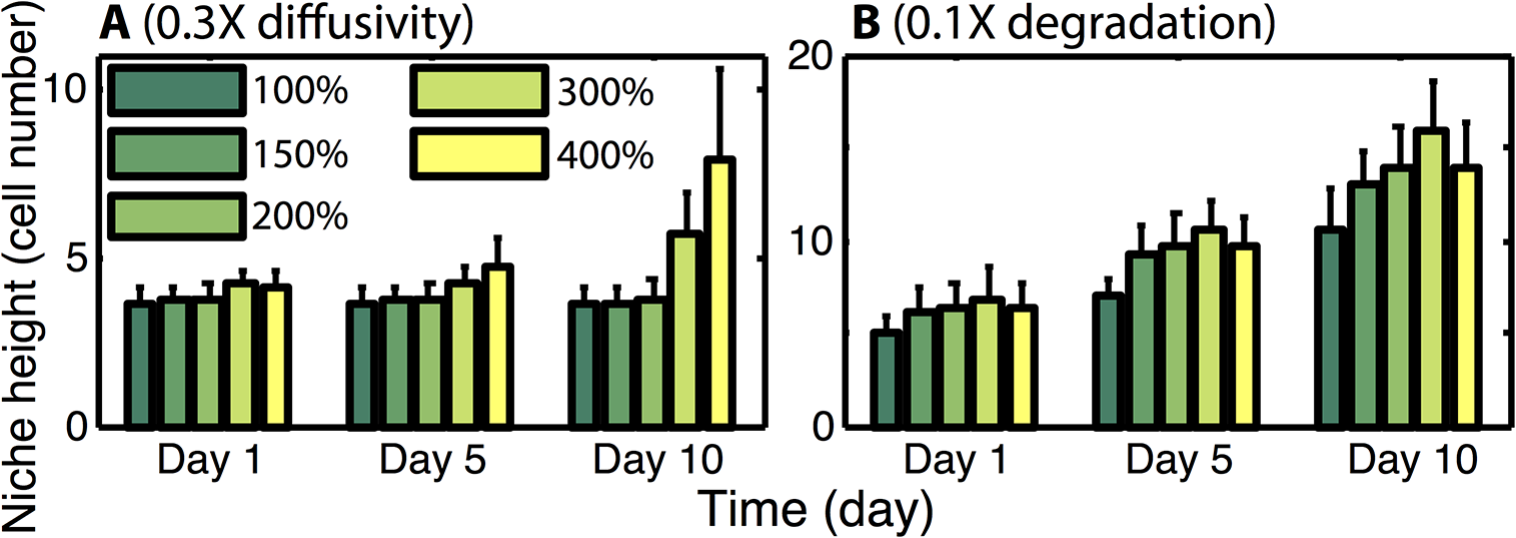
Diffusivity and degradation influence stem cell niche stability. Panels A and B show the effect of reducing diffusivity or the strength of local Wnt degradation on niche dynamics. 0.3X and 0.1X each indicate the factor by which the relevant parameter is reduced. In A it is “*D*” that is reduced while in B, “*d”* is reduced.

From this, we see that increasing the rate of either diffusion or decay will reduce the Wnt concentration near the production source. As a result, we would expect either of these manipulations to have the effect of repressing niche expansion, while also reducing the ability of Paneth cells to maintain the niche on their own. Alternatively, we would expect reducing either would promote expansion and simulation results where both manipulations are performed (Fig 5) do show these manipulations promote expansion. Thus, in addition to the production rate of Wnt, its diffusion and degradation also have an important influence on homeostasis. More specifically, these properties determine whether local concentrations (near the source of production) are sufficiently high to sustain the positive feedback between stem and Paneth cells that drives expansion.

#### Noise in Wnt and BMP affect niche stability to cause expansion

Previous results were obtained under the assumption that gradients of Wnt and BMP activity were fixed, perfectly linear gradients that don’t vary in time. In reality, these gradients are inherently noisy, varying in both time and space due to fluctuations of their synthesis, noisy local environment, and noisy biochemical events through interacting with other components that may fluctuate as well. We thus next ask how this noise influences homeostasis. To do so, we follow the same simulation protocols as before but instead assume these signaling gradients are linearly graded with a superimposed multiplicative noise term. For simplicity, we assume this noise is spatially uncorrelated, so that the noise variations at any two locations are independent. Since we do not have access to estimates of the size of these noise variations, we initially assume each exhibits a similar degree of stochasticity and consider the influence of noise amplitude on crypt dynamics.

**Fig 6:**
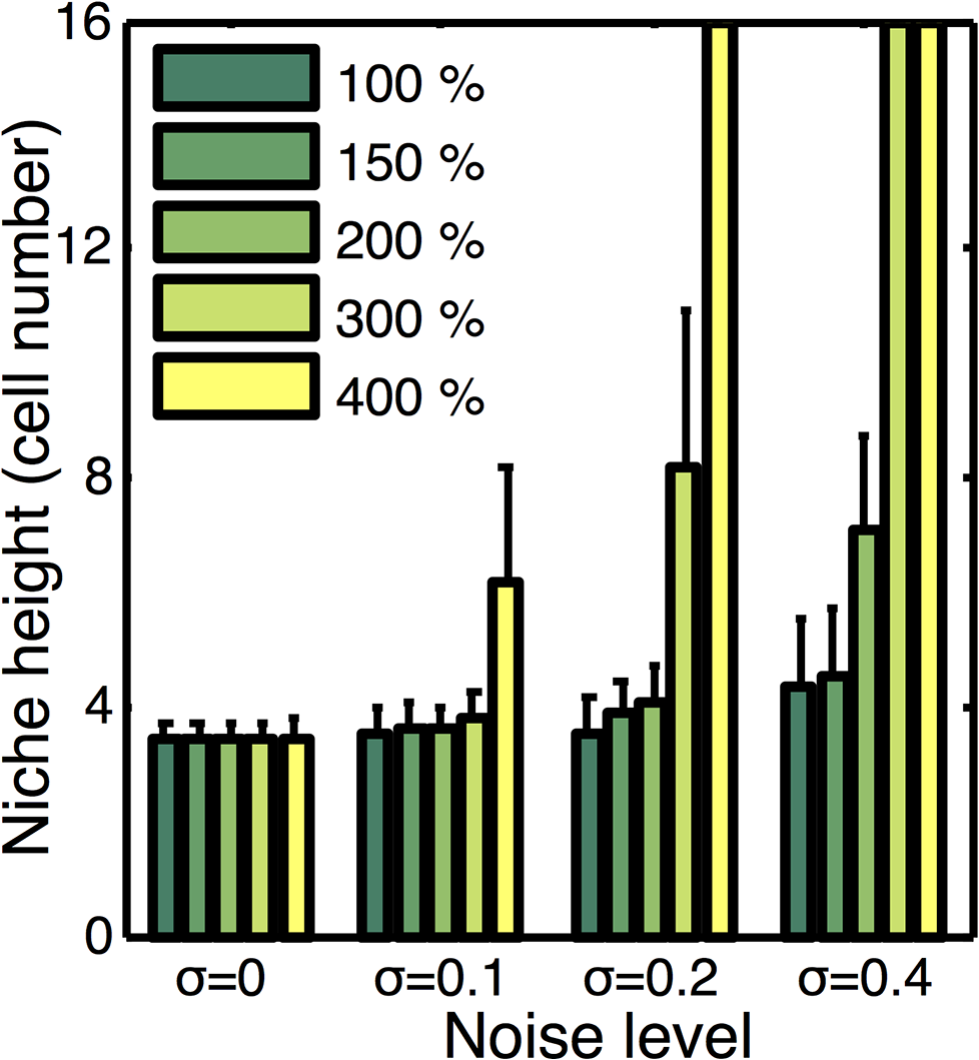
Influence of signaling noise on niche dynamics and stability. Plot of stem cell niche height at steady state as a function of Wnt production rate and the amplitude of imposed noise (*σ*). Noise levels for the exogenous Wnt and BMP gradients are considered to be similar, so in each case we consider each to have noise amplitude of 0, 0.1, 0.2, and 0.4 respectively. Mean and standard deviation over an ensemble of 10 simulations at each production level and noise amplitude is reported. Cases where the bar extends to the top indicate the niche is unstable and expands to occupy the entire crypt.

Simulation results indicate that as expected, more noise leads to a higher degree of variation in the size of the stem cell niche; the standard deviation bars become larger in Fig 6 as noise amplitude increases. Additionally, there is a general trend that increased noise amplitude leads to a larger niche. However, this expansion is marginal and for moderate Wnt production rates and noise levels (e.g. 200% with *σ* = 0–0.2), this noise has little influence on the niche. Surprisingly we find that under more extreme circumstances (both high noise levels and Wnt production rates), that noise has a profound influence on dynamics. Consider the 400% Wnt production rate in Fig 6. When noise levels are low, the stem cell niche expands, but remains stable. When noise levels are further increased however, that expansion becomes uncontrolled and the niche takes over the entire crypt. Thus noise at sufficiently high levels, coupled with high Wnt production rates, can destabilize an otherwise stable crypt.

We further considered the influence of noise in BMP and Wnt individually. To do so, we allowed the noise in these two gradients to be different, and screened over different combinations of noise sizes (results not shown). We found that the two sources had essentially the same influence on dynamics. That is, they do not interact in either positive or negative ways. The likely cause of this is the fact that they influence different cellular properties. BMP influences proliferation while Wnt influences differentiation.

These results show there are two basic regimes of behavior where the influence of noise differs. For all but the most extreme circumstances (both high Wnt production rate and large noise variations), the niche is stable and robust against noise fluctuations. In extreme circumstances however, stochasticity can completely destabilize the crypt. While we do not have measurements to constrain noise levels and Wnt production rates *in vivo*, we can make some basic inferences. While frequent “measurements” of these signals could potentially have significant stochasticity, the processes that are influenced by these signals (proliferation and differentiation) occur on longer timescales (minutes to hours) than typical noise processes (seconds). Thus, the cells measurement of these signals would likely involve some form of temporal integration or averaging that would naturally reduce noise variations. Further, as discussed previously, Wnt production levels at the 150–200% levels are sufficient to supply the system with redundancy (Fig 3B). At these levels, Paneth cell derived Wnt can completely sustain the niche if the global gradient is removed or perturbed. Further, higher levels introduce additional negative effects such as an enlarged niche. Thus, it would not be necessary to produce Wnt at the high rates that interact negatively with noise. So while noise can have a substantial negative effect on crypt dynamics, there is a substantial operating regime where the combination of BMP and locally produced Wnt would maintain a stable crypt that is robust against perturbation or even loss of mesenchyme Wnt, as well as noise in the Wnt or BMP signals.

## Discussion

The murine intestinal crypt epithelium is one of the most dynamic organs in the body, completely replenishing itself every 4–5 days. This quick turnover improves epithelium integrity in the intestinal environment where cells are under constant assault from toxins, gastric acids, and microorganisms. The speed of this replenishment and the fact that this continually occurs over the life of an organism however raises the question, how are size and structure robustly maintained. It is well known that a pool of approximately 10–15 fast cycling crypt base columnar “stem” cells (CBCs) at the base of each crypt is responsible for constant renewal [7,8,12]. But while a number of molecular regulators that influence CBC proliferation and differentiation dynamics have been identified, it remains unclear how these regulators coordinate to maintain homeostasis.

Here, we construct a discrete, multiscale model of the evolving crypt and interrogate the role of different hypothesized regulators on homeostasis. This model uses a subcellular element formalism [49] to describe the structure of cells, their interactions, and their interactions with the crypt wall. On top of this formalism, which primarily describes physical aspects of the system, we include the dynamics of cellular commitment, cell-cell signaling (Notch signaling in particular), and the presence of diffusible signals that influence cellular commitment. We do note that there are a number of aspects of crypt biology that we do not account for. In particular, there are at least seven different cell types present in the crypt, some of which we do not include. We do not account for the polarized nature of cells [58,59], the resulting function of those cells (e.g. transport of material into and out of the crypts lumen), or systemic responses to damage [60]. Similarly, we do not account for density dependent inhibition of proliferation or damage-induced effects such as activation of +4 cells [10,11]. The goal of this exposition is to investigate the role of different regulatory mechanisms in maintaining homeostasis in healthy crypts. Toward this goal, we incorporate the features of crypt biology most germane to this context and leave the inclusion of these additional features for future work. In particular, we use this model as a platform to investigate the influence of 1) Paneth cell derived Wnt, 2) BMP signaling, and 3) Ephrin mediated repulsion of Paneth cells on homeostasis as well as 4) the influence of signaling noise on crypt structure.

It is well established that continual activation of the canonical Wnt signaling pathway is required to prevent stem cell differentiation. However, there are multiple sources of Wnt signaling [2]. First, the mesenchyme surrounding the crypt generates a Wnt gradient that is highest at the base. It has been shown previously that this gradient in concert with Notch lateral inhibition and Paneth cell migration (driven by Ephrin signaling) can maintain a crypt with the proper structure [42]. However recent evidence suggests that Wnt secreted by Paneth cells, which are interleaved with CBCs at the base, is sufficient to both generate and maintain crypts [20], suggesting redundancy. Our results suggest that these two Wnt sources have functionally different effects on crypt homeostasis, and that the influence of this additional Wnt source depends critically on its rate of production. When that rate is below a critical level, it is not sufficient to maintain the niche and exogenous Wnt is required. In this case, it has no influence on homeostasis. If on the other hand, the production rate is above this critical level, the crypt becomes robust against perturbations or even complete removal of the exogenous Wnt source.

This redundancy however comes at an expense. This source of Wnt combined with the mutually reinforcing feedback between stem and Paneth cells creates a positive feedback that drives uncontrolled expansion of the niche, at the expense of the remainder of the crypt. Further results however show that BMP signaling, which forms an opposing gradient to the exogenous Wnt gradient and inhibits proliferation [26,28] constrains this expansion and promotes homeostasis. Results also suggest a different possible explanation for the downward migration of Paneth cells. It is commonly held that this motion is required to maintain the niche. Simulation of a number of different model variants however suggests that abrogation of this migration does not destroy the niche. On the contrary, removal of this motion in some circumstances leads to its uncontrolled expansion, even when BMP inhibition is present. Thus rather than being required to maintain the niche, this motion may instead be required to constrain its expansion and maintain the remainder of the crypt.

There is a caveat to this result of course. In this investigation we have assumed that Paneth cell function is independent of location in the crypt. That is, they can secrete Wnt at any location in the crypt. It is possible that Paneth cell function is inherently tied to location though, i.e. they can only secrete Wnt if they are at the base. This would however create an entire different regulatory mechanism. In this case, Paneth cells would essentially act as amplifiers of the external Wnt gradient. In this way, they would not be participating in an auto-regulatory feedback loop but rather would be an intermediary of a purely external regulatory scheme. We do not reject this possibility, but do suggest it is seemingly inconsistent with *in vitro* “mini guts” results. In that setting, there is no external tissue or signal telling Paneth cells where they are. Furthermore, they are the only source of Wnt, suggesting they act as more than a passive amplifier of external Wnt signals and do participate in an auto-regulatory feedback.

We further investigated the robustness of a niche regulated in this manner to noise in exogenous Wnt and BMP signals. Results show that as expected, noise has the effect of introducing a small amount of variability in the size of the niche. More unexpectedly, we find that the introduction of noise actually increases the size of the niche. In most cases, this is not significant and for all practical purposes, the niche is robust against these noise sources. In extreme circumstances however, where both noise levels and the rate of Wnt production are quite large, this noise actually destabilizes the niche causing uncontrolled expansion. These extreme circumstances however appear to be outside the physiological regime, and we thus conclude that there is a sizable operating regime where a regulatory system of this form creates a homeostatic environment that is relatively insensitive to noise in Wnt and BMP.

The essential biological feature that gives rise to these results is that Paneth cells create a “mini niche” surrounding them. While this is well characterized experimentally [21], the implication of this to crypt stability and homeostasis, which is the topic of this investigation, is relatively less understood. The central result here is that these “mini niches” form a local, auto-regulatory feedback loop that supplements external Wnt signaling to redundantly reinforce crypt renewal.

This hypothesis leads to a few predictions. First, when Paneth derived Wnt levels are sufficient to maintain the niche, removal of BMP is predicted to lead to aberrant expansion of the niche. Previous results have indeed shown that deletion of BMP signaling leads to crypt fission [29] and the formation of ectopic crypts [61], which is one potential effect of this expansion. An alternative prediction is that the *in vivo* crypt can be maintained in the absence of mesenchyme derived Wnt. Testing this prediction would require deleting mesenchyme derived Wnt without perturbing Paneth cell derived Wnt or the β-catenin required to transduce Wnt signals. Fevr et al. [17] demonstrated that Wnt deletion leads to terminal differentiation of all crypt stem cells. This investigation however deleted cell’s ability to transduce all Wnt signals. It has been verified *in vitro* that external Wnt signals are not required for crypt development or maintenance, however these results are confounded with the presence of additional Wnt regulators, chiefly R-spondin. A sounder test of this regulatory scheme requires specific removal of the exogenous Wnt signal *in vivo*, without perturbing the proposed auto regulatory feedback.

These results paint a somewhat different picture of small intestinal crypt homeostasis from the existing view. In the canonical view, Wnt is a master regulator that serves as a morphogen of sorts, creating a road map that links cell properties to their locations. This however implicitly assumes the niche is a delicate environment that needs to be supported (by Wnt). Results here suggest that instead, auto-regulation pushes the niche into a constant state of expansion and that various forms of negative regulation constrain that expansion. This hypothesis is consistent with observations in other systems, such as the olfactory epithelium or the hair follicle niche, where negative regulation is a critical component of niche dynamics. More generally, these results are in line with observations in other systems showing that cells of a niche actively participate in the maintenance of their own microenvironment, rather than being slaved to external regulation.

## Materials and Methods

### Subcellular element model

Each individual cell is represented by a set of *N* connected elements. *N* is chosen to be 20 in simulation to balance between the flexibility of modeling cellular dynamical activity and computational costs. The dynamics of each element is determined by biomechanical forces, which consist of intracellular interaction among the elements of the same cell, intercellular interaction between elements of different cells, and external force due to environmental cues. The equation of motion of the position vector $Y_{\alpha_{i}}$ of element *α*_*i*_ for cell *i* is

$$\frac{dY_{\alpha_{i}}}{dt}=-\nabla_{\alpha_{i}}\sum_{\alpha_{i}\neq \beta_{i}}V_{intra}\left(\left|Y_{\alpha_{i}}-Y_{\beta_{i}}\right|\right)-\nabla_{\alpha_{i}}\sum_{i\neq j}\sum_{\beta_{j}}V_{inter}\left(\left|Y_{\alpha_{i}}-Y_{\beta_{j}}\right|\right)-\nabla_{\alpha_{i}}F_{external}\left(Y_{\alpha_{i}}\right),$$

where *V*_*intra*_ is a pairwise force interaction between elements *α*_*i*_ and *β*_*i*_ of the same cell *i*, *V*_*inter*_ is a pairwise force interaction between elements *α*_*i*_ of cell *i* and *β*_*j*_ of cell *j*, and *F*_*external*_ is an external force representing membrane adhesion and other environmental interactions.

All elements within a cell interact according to the spring potential [47,48]

$$V_{intra}=\mu \frac{{\left(r_{ij}-r_{0}\right)}^{2}}{2},$$

where *r*_*ij*_ is the distance between element *i* and element *j* of the same cell and *r*_0_ is a rest length. In the absence of external forces, the intra-cellular forces will scatter the inner elements to the minimum energy configuration with a roughly spherical shape of preferred size. That size is determined by the rest length *r*_0_ for *V*_*intra*_, defining a volume of sorts for the cell.

The inter-cellular force interactions are described by Lennard-Jones type potentials [47,48]

$$V_{inter}=\varepsilon \left({\left(\frac{\sigma }{\left|r_{ij}\right|}\right)}^{12}-{\left(\frac{\sigma }{\left|r_{ij}\right|}\right)}^{6}\right)$$

where *r*_*ij*_ is the distance between element *i* and element *j*. The parameter *ε* determines the strength of interaction. *σ* is the equilibrium separation where the inter-element potential is zero and two elements are at relative balance position. If the distance between two elements is smaller than *σ*, they experience a repulsion force to prevent overlap of the cell bodies. When the distance between the elements is greater than *σ*, but less than a cutoff value, an attraction exists between the elements. Beyond this cut-off value, we assign zero interaction between cells. These medium range interactions are designed to represent the surface interactions of cadherin-mediated cell-cell adhesion.

We consider a simplified crypt structure with a test tube geometry (similar to [42,48]), which is a cylinder attached above a semi sphere. The tube is chosen to have 16 cell diameter in height and 6 cell diameter in diameter. The adherent force between cell elements and the crypt wall is defined by

$$F_{external}(Y_{\alpha_{i}})=\frac{\varepsilon_{external}}{\left|r_{i}\right|}.$$

Here, *ε*_*external*_ is the strength of external force, and *r*_*i*_ is the distance between element *i* and the crypt wall. This force has a cut-off distance of half the rest diameter of a cell, to ensure that only the elements “attached” to the wall experience the attraction.

The friction between cells and the basal membrane is modeled as a linear drag with the equation $F_{d}=b_{z}\vec{v}$, where *b*_*z*_ is the linear drag constant in z-axis and $\vec{v}$ is the velocity of the cell. The force of drag is approximately proportional to velocity, but opposite in direction to mimic the rupture of many ligand bonds distributed on the cell membrane.

### Coupling of subcellular element model and chemical diffusion equation

To couple cell dynamics and signaling pathway, a regular, rectangular grid for chemical diffusion is superimposed on the subcellular element model domain. Each simulation time step for the evolution of the full system consists of a substep of subcellular element model followed by a substep evolving the state of the chemical field according to the reaction diffusion PDE. During the substep of the cell-based subcellular element model, cells move to a new location, undergo growth and division, make lineage decisions, and produce Wnt signals, which modifies the local Wnt field. Each Paneth cell serves as a Wnt source. This is implemented by having each Paneth cell element secrete Wnt at a rate *δ*_*c*_. This production is extrapolated from the element’s position to the nearby grid elements, so that this production acts as a distributed source in the chemical diffusion PDEs. Each cell in turn reads the Wnt concentration at its location to make fate commitment decisions. This is implemented using standard trilinear interpolation where the concentration value at each element position is determined from the chemical field on the regular grid. Subsequently, the value of the external Wnt field is added to this value to produce the total Wnt concentration. For each cell, a linear combination of the Wnt concentration at each of its elements is then used to determine the net Wnt concentration that cell is exposed to.

During the substep of chemical diffusion evolution, not only do Paneth cells serve as sources of Wnt production, but all cells serve as barriers for diffusion. If a well-defined boundary of each cell were determinable, diffusion could simply be prohibited within those boundaries. However using this methodology, cells do not have a well-defined boundary but instead are made up of elements. To mimic the restriction of diffusion to the exterior of the cells, we make the rate of diffusion dependent on the local number density of elements. That is, for each grid node, the number of elements in neighboring grid spaces is computed, with the rate of diffusion decreasing as this quantity increases. Diffusion is also restricted to the epithelium itself under the assumption that the basement membrane is impermeable. To implement this, we expand the computational grid beyond the crypt itself so that a regular, rectangular grid can be employed. We then assign a zero diffusion coefficient at nodes beyond the crypt walls.

To solve Eq (1), we apply a second-order central difference for the spatial derivatives, and a forward Euler scheme to the temporal discretization. Step size in space is chosen to be 1 *μ*m. For each step of chemical diffusion evolution, the chemical field is updated for 1000 times, giving dt = 0.0036s for chemical equation updates.

### Generating a crypt initial condition for simulations

To build up the initial configuration of a crypt, we begin with a test simulation where only two stem cells placed at the crypt bottom. These cells (which have no identity at this point) are allowed to proliferate and move until the daughter cells cover up the whole crypt to form a compact cell packing. Cell Notch levels and identities are then initialized with the canonical spatial cell distribution: stem and Paneth cells interleaved with each other at the bottom of crypt with enterocytes and Goblet cells residing in the top of the crypt. The inner time counter for each cell is then chosen as a random number smaller than its pre-assigned life cycle to initialize each cell’s cycle length.

### Determining the critical production rate of local Wnt

The chemical diffusion-reaction equation is given by Eq (1) in the Results section. Estimates for *D*, *d* were obtained from [26,62] (Table 1). However we do not have an estimate for either the Wnt concentration *in vivo* or the rate of Wnt secretion rate by Paneth cells. It is known however that in this system, Wnt is lapidated and thus has a short diffusion length scale [2]. We thus assign a base value of the Wnt production rate as that rate at which the Wnt threshold *TH*_*Wnt*_ is achieved at a distance of 12.5 microns from the source (the equivalent of 1.25 cell diameters). This is chosen to represent a type of threshold production rate. Above this rate, local Wnt concentrations will presumably be sufficiently large to maintain a stem cell in near direct contact. Below this rate, it will not. We compute this critical rate *δ*_*c*_ by considering a simplified setting where a single Paneth cell is placed at *x* = 0 in a one-dimensional domain. The concentration field is then simulated for a range of values and the critical value at which this condition is achieved is recorded.

### GPU implementation

The most computationally intensive component of this method is computation of the forces between elements that drive cell motions. This is an N-body simulation where the force between all pairs of elements must be computed. This scales as *O*(*n* ^2^) where *n* is the number of subcellular elements in the system. Fortunately, this computation is also highly parallel and suitable for GPU implementation. We followed [49] to provide a parallel implementation of the subcellular element model, and include memory layout of data structures and functional decomposition for efficient implementation. In this implementation, the highly-parallel parts like force and dynamic computations, which do not require dynamic updating of data structures, were carried out on the GPU using OpenCL. The less intensive computations such as cell division and growth, which require updating data structures, were carried out on the CPU using C. To minimize memory transfer between the GPU and CPU, a fixed number of cell position updates are iteratively computed on the GPU and the data is subsequently shipped to the CPU where a single growth / division / differentiation update is performed. The data is then transferred back to the GPU where subsequent updates are performed and this process is iterated over the length of the simulation.
